# Association between obesity type and dyslipidemia by age and sex: a cross-sectional study using data from the 2022 Korea National Health and Nutrition Examination Survey

**DOI:** 10.7586/jkbns.25.019

**Published:** 2025-05-26

**Authors:** Eun-Hye Lee, Yun-Hee Kim

**Affiliations:** Department of Nursing, Pukyong National University, Busan, Korea

**Keywords:** Obesity, Dyslipidemias, Body mass index, Body composition

## Abstract

**Purpose:**

Obesity is a well-established risk factor for dyslipidemia. Normal-weight obesity (NWO) is associated with lipid profile abnormalities. However, research in Korea on the relationship between NWO and dyslipidemia is limited. Therefore, this study evaluated the relationship between obesity types, including NWO, and dyslipidemia, and investigated these relationships by age and sex.

**Methods:**

Secondary data analysis was performed using data from the 9th Korea National Health and Nutrition Examination Survey conducted in 2022. In total, 3,805 participants were analyzed using complex sample multiple logistic regression based on body mass index and body fat percentage (BF%).

**Results:**

NWO was significantly associated with an increased risk of dyslipidemia relative to normal weight non-obesity (odds ratio: 1.66, 95% confidence interval: 1.34~2.06). Notably, individuals with NWO under the age of 65 and men had a higher risk of dyslipidemia, underscoring the importance of targeted health management in these populations.

**Conclusion:**

NWO was positively correlated with dyslipidemia. Therefore, the role of clinical nurses in early screening for NWO is of considerable importance, particularly among high-risk populations such as men under the age of 65. To support obesity management and the prevention of dyslipidemia, nursing interventions aimed at reducing BF% by promoting lifestyle modifications and regular monitoring are essential.

## INTRODUCTION

Dyslipidemia serves as a key determinant in cardiovascular disease (CVD), in which changes in blood lipid levels contribute to atherosclerosis, leading to the development of CVD [1]. The number of deaths from CVD is increasing globally [2], and a similar upward trend has been observed domestically in Korea [3]. Recent data from the Korea Disease Control and Prevention Agency (KDCA) on CVD incidence indicate that the number of individuals diagnosed with CVD has continued to increase. Specifically, cases of myocardial infarction rose by 54.5% from 2011 to 2021, while stroke cases increased by approximately 9.5% during the same period [4]. According to the Dyslipidemia Fact Sheet 2024 of the Korean Society of Lipid and Arteriosclerosis, the prevalence of dyslipidemia in adults between 2016 and 2022 was 47.4%, with a tendency to increase with age [5]. Despite its high prevalence, dyslipidemia remains under-recognized and inadequately managed compared to hypertension and diabetes mellitus, resulting in insufficient disease control [6,7]. Thus, recognizing the risks associated with dyslipidemia and adopting proactive management strategies are essential for effective CVD prevention.

Obesity is more strongly associated with dyslipidemia than other risk factors [8,9]. Obesity, characterized by excessive body fat accumulation, disrupts lipid-protein metabolism in lipoproteins, leading to dyslipidemia [9,10]. Previous studies have identified body fat content as a significant predictor of hyperlipidemia [11]. Globally, the prevalence of obesity has more than doubled since 1990 [10], and domestic obesity rates have consistently risen from 30.9% in 2014 to 37.2% in 2023, with variations across age and sex [12-14]. Between 2014 and 2023, obesity rates increased by 7.8% in men and 4.5% in women. The rise was even more pronounced among younger adults, with increases of 9.7% in their 20s, 8.0% in their 30s, and 6.6% in their 40s, highlighting a sharper upward trend in men and younger age groups [12]. Given the significant prevalence of obesity, early screening and comprehensive management of affected individuals are vital to effectively mitigate dyslipidemia associated with this condition.

There are various methods for classifying types of obesity. Body mass index (BMI) is one of the frequently utilized methods for diagnosing obesity, however, it has limitations in accurately determining obesity because it does not differentiate between fat and muscle [15]. In contrast, incorporating body composition analysis enables the measurement of body fat percentage (BF%) and identification of normal-weight obesity (NWO) [15-17].

NWO can be described as a situation where BMI remains within normal limits but BF% is elevated [16,17]. Although individuals with NWO may appear to have a normal physique [18], they are characterized by abnormal blood lipid profiles [16,17]. The global prevalence of NWO is estimated to range from 4.5% to 22% [19], owing to its high prevalence, several international studies consider NWO as a type of obesity [20]. Domestically in Korea, its prevalence is even higher at 32% [21]. However, recent domestic obesity-related studies have failed to adequately consider NWO and have primarily evaluated obesity based on BMI alone [22]. NWO is comparable to obesity in terms of body composition and fat distribution [17,23], therefore, health management for this condition is equally as critical as that for obesity.

However, NWO is typically associated with a lack of awareness regarding health issues and the misconception that there are no health problems, leading to a greater risk of neglecting health management [18]. Therefore, early screening of individuals with NWO and the implementation of effective obesity-related nursing interventions are necessary.

Moreover, a review of previous domestic studies indicates that research on the relationship between NWO and dyslipidemia is limited [24-26]. Most domestic research has either focused on specific population groups or stratified participants by sex, and few studies have stratified the general adult population using 65 years as a cutoff point [25,26]. According to previous studies, blood lipid levels vary by age and sex [13]. Although the prevalence of dyslipidemia tends to increase with age, it demonstrates a declining trend after the age of 65 [13].

This study aimed to use nationally representative big data from the Korea National Health and Nutrition Examination Survey (KNHANES) to classify obesity type, including NWO, and to analyze their association with dyslipidemia based on age and sex. Additionally, this study highlights the necessity of addressing NWO as a critical focus in obesity management and aims to provide foundational data to guide effective nursing interventions. Collectively, through these efforts, this research underscores the significance of health management related to NWO and aids in informing strategic directions for future nursing practices.

## METHODS

### 1. Research design

This study employed a cross-sectional design and conducted a secondary data analysis of the 2022 (1st year) data from the 9th KNHANES to investigate the relationship between obesity type and dyslipidemia according to age and sex among Korean adults.

### 2. Participants

**Figure 1.**
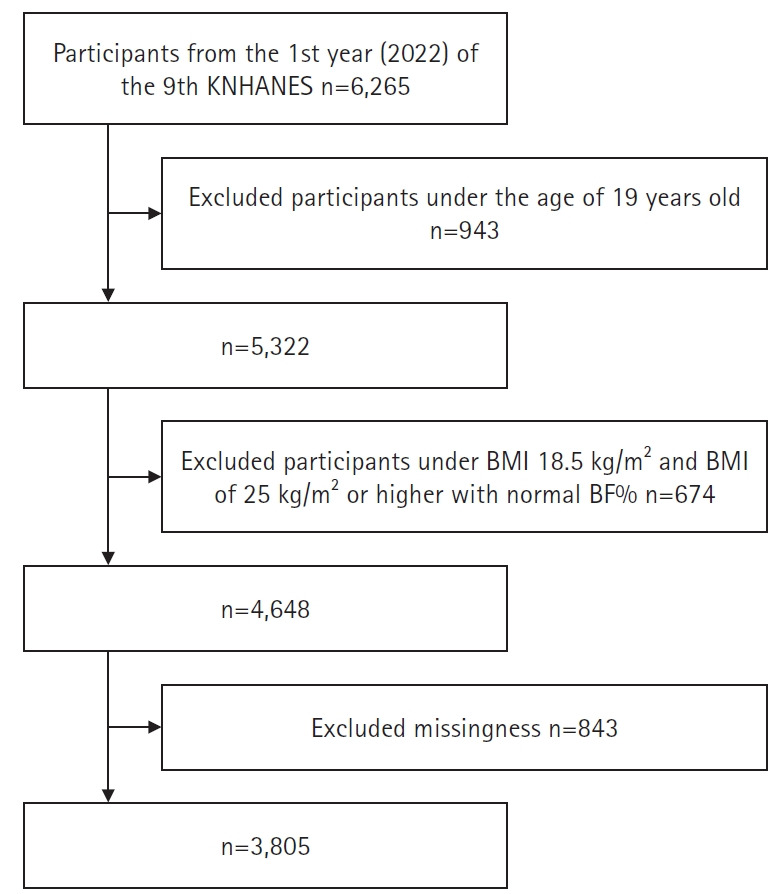
Flow chart of the study protocol. KNHANES = Korean National Health and Nutrition Examination Survey; BMI = Body mass index; BF% = Body fat percentage.

This study was conducted using integrated health survey and health examination data from the 1st year (2022) of the 9th KNHANES. The study population consisted of the entire national population, and a multistage stratified cluster sampling method was used to select 25 households per cluster from 192 survey areas, with a total of 6,265 participants. After excluding individuals under 19 years of age, those with a BMI under 18.5 kg/m^2^, and individuals with a BMI ≥ 25.0 kg/m^2^ and a normal BF% according to sex-specific criteria. And, excluding individuals who had missing data, 3,805 participants were included in the final analysis (Figure 1).

### 3. Instruments

#### 1) Demographic characteristics, health behaviors, and chronic disease-related characteristics

The demographic characteristics included age and sex, with age categorized as under 65 years (19~64 years) and over 65 years.

Health behaviors included smoking, drinking, sleeping duration, sitting time, aerobic physical activity and subjective health status. Smoking status was classified according to current cigarette use, where individuals who reported that they 'formerly smoked but do not smoke now' or 'never smoked in their lifetime' were categorized as 'No' and those who reported 'smoking daily' or 'smoking occasionally' were classified as 'Yes'. Drinking was classified based on the frequency of drinking over the past year, with individuals who reported 'never drinking' or 'drinking less than once a month' categorized as 'No' and those who drank 'from once a month to four or more times a week' categorized as 'Yes'. Sleep duration was categorized by the average weekday sleep time, with individuals sleeping 'at least 7 hours' or 'less than 7 hours' [27]. Sitting time was classified by the average daily sitting time on weekdays, with categories for 'less than 5 hours' and '5 or more hours' [28]. Aerobic physical activity was classified as 'Yes' for individuals who engaged in at least 2 and a half hours per week of moderate-intensity exercise, at least 1 hour and fifteen minutes per week of vigorous-intensity exercise, or a combination of both moderate- and vigorous-intensity exercises. Those who did not meet these criteria were categorized as 'No'. Subjective health status was categorized by self-reported health perception, where those who evaluated their health as 'bad', 'very bad' were categorized as 'Bad' and those who evaluated their health as 'very good', 'good', 'fair' were categorized as 'Not bad'.

Chronic disease-related characteristics included hypertension and diabetes. Hypertension was classified based on the hypertension diagnostic criteria, the consumption of antihypertensive medication, or a physician’s diagnosis [29]. Diabetes mellitus was classified based on a fasting blood glucose level of ≥126 mg/dL, use of antidiabetic medication, or a physician’s diagnosis [29].

#### 2) Obesity type

Obesity was classified based on a combination of BMI and BF%. BMI is a derived variable calculated from anthropometric measurements of height and weight, and BF% was measured using a bioelectrical impedance analyzer (Inbody 970, Biospace, Korea). BMI was grouped into normal weight and obesity according to the World Health Organization Asia-Pacific obesity criteria and previous studies [15,30]. The BF% thresholds (20.6% for men and 33.4% or more for women) followed the NWO criteria proposed in a study based on the KNHANES [21]. These thresholds were identified as the optimal values for predicting CVD risk factors through receiver operating characteristic analysis, with sensitivity and specificity maximized using Youden’s Index [21]. Normal weight non-obesity (NWNO) was defined as BMI of 18.5~24.9 kg/m^2^, with a BF% of ≤20.6% for men and ≤ 33.4% for women. NWO was defined as BMI of 18.5~24.9 kg/m^2^, with a BF% of ≥ 20.6% for men and ≥ 33.4% for women. Obesity is defined as BMI ≥ 25.0 kg/m^2^, with a BF% of ≥ 20.6% for men and ≥ 33.4% for women [21,30].

#### 3) Dyslipidemia

Dyslipidemia was characterized by the presence of at least one of the following: hypercholesterolemia (total cholesterol ≥ 240 mg/dL), hypertriglyceridemia (triglyceride ≥ 200 mg/dL), hyper-low density lipoprotein (LDL)-cholesterolemia (LDL-C ≥ 160 mg/dL), hypo-high density lipoprotein (HDL)-cholesterolemia (HDL-C < 40 mg/dL), taking dyslipidemia medication, or a physician's diagnosis [24].

### 4. Data analysis

In this study, the complex sampling method reflecting the raw data characteristics was used to analyze the statistical data from the KNHANES. The analysis used SPSS version 27.0 (IBM Corp., Armonk, NY, USA), considering stratification, clustering, and weighting provided by the KDCA to increase the representativeness and accuracy of the variable estimates. The entire set of variables included in the study was categorical variables, and some of the continuous variables were converted to categorical for analysis. The differences in dyslipidemia prevalence were assessed by demographic characteristics, health behaviors, and chronic disease-related characteristics using a χ^2^-test. The relationship between obesity type and dyslipidemia was analyzed using complex sample multiple logistic regression analysis. In Model 1, no variables were controlled. Based on previous studies indicating that blood lipid levels vary significantly according to age and sex [13], and stratified analysis was conducted to more accurately assess the impact of age and sex on the results. Therefore, in Model 2, stratification by age groups (< 65 years, ≥ 65 years) was performed to clearly assess the impact of age on dyslipidemia, and all variables except age (sex, smoking, drinking, sleep duration, sitting time, aerobic physical activity, subjective health status, hypertension, diabetes mellitus) as the stratification variable, were controlled. In Model 3, stratification by sex was conducted to evaluate differences in outcomes according to sex, with men and women analyzed separately, and all variables except sex (age, smoking, drinking, sleep duration, sitting time, aerobic physical activity, subjective health status, hypertension, diabetes mellitus) were controlled.

### 5. Ethical consideration

Approval was obtained from the Institutional Review Board at the Pukyong National University (IRB No. 2025-02-002), this study complied with KDCA's KNHANES raw data usage protocols, including a user agreement and security pledge. The KNHANES data provided was de-identified and cannot be used to identify individuals, ensuring that privacy protection and confidentiality were maintained throughout the research process.

## RESULTS

### 1. Characteristics of participants

The participants included 1,312 NWNO and 1,135 NWO and obesity 1,358, for a total of 3,805 participants. Analysis of dyslipidemia prevalence by obesity type showed rates of 29.2% for NWNO, 46.3% for NWO, and 59.0% for obesity, with both NWO and obesity exhibiting higher prevalence rates than NWNO (χ^2^ = 244.25, *p* < .001). The prevalence was 41.6% in individuals under 65 years and 61.2% in those 65 years and older, with the latter group showing a higher prevalence (χ^2^ = 88.63, *p* < .001). Regarding sex, the prevalence was 40.3% in women and 49.8% in men, with men having a higher prevalence (χ^2^ = 34.57, *p* < .001).

Among other characteristics, significant differences were found in variables such as smoking (χ^2^ = 17.77, *p* = .001), drinking (χ^2^ = 9.08, *p* = .012), aerobic physical activity (χ^2^ = 25.98, *p* < .001), subjective health status (χ^2^ = 35.77, *p* < .001), hypertension (χ^2^ = 258.77, *p* < .001), and diabetes mellitus (χ^2^ = 198.13, *p* < .001), sleep duration, and sitting time showed no significant differences (Table 1).

### 2. Relationship between obesity type and dyslipidemia by age and sex

The association between obesity type and dyslipidemia was analyzed with NWNO set as the reference group. In Model 1, no variables were controlled. NWO adjusted odds ratio (aOR) was 1.66 (95% confidence interval [CI], 1.34~2.06) and obesity aOR was 2.20 (95% CI, 1.79~2.69) showing a statistically significant difference compared to the NWNO group. These results suggest that both NWO and obesity are associated with an increased likelihood of dyslipidemia compared to the NWNO group, with obesity showing a stronger association.

In Model 2, all variables except age (sex, smoking, drinking, sleep duration, sitting time, aerobic physical activity, subjective health status, hypertension, diabetes mellitus) were controlled, in individuals under 65 years of age NWO aOR was 1.99 (95% CI, 1.56~2.54) and obesity aOR was 2.48 (95% CI, 1.97~3.11) showing a statistically significant difference compared to the NWNO group. These results indicate that in individuals under 65 years of age, both NWO and obesity are significantly associated with an increased risk of dyslipidemia. However, no significant difference was observed in individuals aged 65 years and older.

In Model 3, all variables except sex (age, smoking, drinking, sleep duration, sitting time, aerobic physical activity, subjective health status, hypertension, diabetes mellitus) were controlled, for men NWO aOR was 2.29 (95% CI, 1.58~3.33) and obesity aOR was 2.72 (95% CI, 1.88~3.94) while for women NWO aOR was 1.39 (95% CI, 1.04~1.86) and obesity aOR was 1.80 (95% CI, 1.39~2.32). These results suggest that, compared to the NWNO group, both NWO and obesity are significantly associated with increased risk of dyslipidemia in both men and women, although the magnitude of association was higher in men (Table 2).

**Figure 2.**
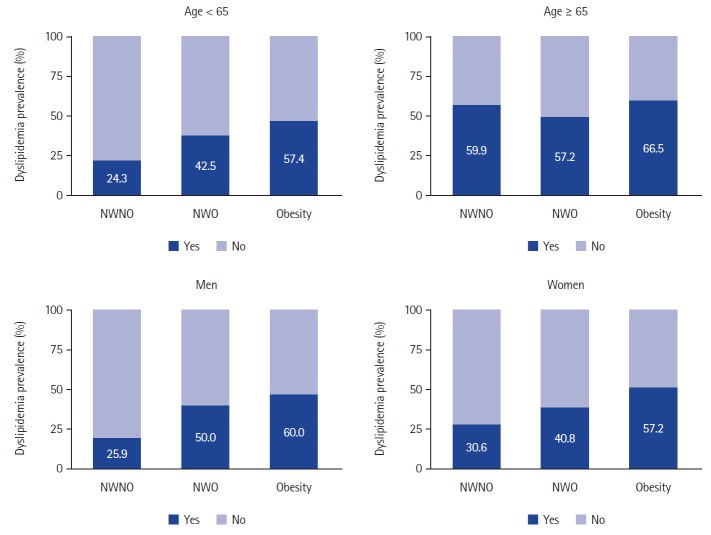
Prevalence of dyslipidemia by obesity type according to age and sex. NWNO = Normal weight non-obesity; NWO = Normal weight obesity.

Dyslipidemia is associated with age and sex [13]. Accordingly, the prevalence of dyslipidemia by obesity type was further analyzed by stratifying the participants into groups of individuals aged < 65 years, ≥ 65 years, men, and women. The results showed that, among individuals under 65 years and in both sexes, the prevalence of dyslipidemia was higher in the NWO group than in the NWNO group, with the obesity group exhibiting the highest prevalence across all age and sex groups (Figure 2).

## DISCUSSION

This study was conducted to analyze the connection between obesity type and dyslipidemia for adults 19 years and older, using raw data from the 9th KNHANES conducted in 2022, based on age and sex.

The prevalence of obesity type in this study was 29.8% for NWO. The rate of NWO exceeds the worldwide prevalence [19]. A domestic study based on KNHANES data from 2008 to 2011 reported an NWO prevalence of 19% [26], indicating a significant increase in prevalence over time. The elevated prevalence of NWO may increase the risk of various health conditions [31,32], however, depending on the obesity diagnosis method, NWO may be excluded from management strategies. Therefore, the inclusion of NWO in obesity-related research could provide a more accurate classification of obesity and improve management strategies. Consequently, it is essential to employ lipid profile and body composition analyses for NWO screening in clinical nursing practice, and it is important to identify NWO early and implement nursing interventions distinct from those used for general obesity.

The prevalence of dyslipidemia differs by obesity type, NWO showed a higher risk of developing dyslipidemia than those with NWNO. Several domestic and international studies have reported that NWO carries a higher risk of dyslipidemia than NWNO [25,33], which agrees with this study's findings. Moreover, since BF% is a key predictor of dyslipidemia [34], it suggests that within the NWO category, a higher BF% may be associated with an increased prevalence of dyslipidemia. Excessive fat accumulation may result in metabolic disturbances and promote the accumulation of atherogenic lipoproteins, ultimately causing dyslipidemia [9]. Therefore, targeted health management strategies such as reducing body fat are essential to prevent dyslipidemia in individuals with NWO.

In addition, significant differences in participant characteristics were observed according to the presence of dyslipidemia. Smoking, physical inactivity, poor subjective health status, and the presence of chronic diseases such as hypertension and diabetes were associated with dyslipidemia. These findings suggest that individuals with dyslipidemia exhibit specific health risk characteristics, indicating the need for tailored interventions targeting comprehensive health behaviors, including lifestyle modification and chronic disease management.

In this study, obesity type was analyzed based on age categories. Individuals under 65 years with NWO had a higher risk of developing dyslipidemia than those with NWNO, indicating a significant effect. On the other hand, no significant relationship was found in individuals aged 65 and older. Previous studies have shown that understanding the relationship between NWO and metabolic disorders is crucial in younger individuals [33]. Correspondingly, another study found that the risk of developing dyslipidemia due to NWO was higher in individuals under the age of 60 compared to those aged 60 and older [35]. These findings suggest that NWO has a greater impact in younger age groups, increasing the risk of dyslipidemia. In contrast, the influence of NWO appeared to diminish with increasing age. The stronger association between NWO and dyslipidemia in younger individuals may be attributed to lifestyle factors such as physical inactivity, smoking, subjective health status, and dietary habits [23,36]. These findings indicate that beyond age differences, lifestyle factors have a significant influence on the connection between NWO and dyslipidemia. Additionally, in individuals aged 65 years and older, other risk factors may have a stronger association with dyslipidemia than BF%. Specifically, fat distribution, particularly abdominal obesity as indicated by waist circumference, along with comorbid conditions such as hypertension and diabetes, appear to play a more prominent role in the development of dyslipidemia in this age group [36,37]. Therefore, future research should systematically analyze the health risks associated with NWO through age-specific management and adjustments in lifestyle habits.

When NWO was analyzed based on sex, men had a higher risk of developing dyslipidemia than women. Several domestic and international studies have similarly reported that men with NWO have approximately twice the risk of developing dyslipidemia as women [26,32], supporting the findings of this study. These sex differences can be further explained by variations in metabolic characteristics related to fat distribution. Visceral fat, which is predominantly accumulated in men, is metabolically active and promotes insulin resistance, systemic inflammation, and dyslipidemia, thereby increasing the risk of CVD. In contrast, women, who tend to have a higher proportion of subcutaneous fat, experience a relatively lower metabolic risk and benefit from a protective effect against the progression of obesity [14]. Furthermore, estrogen activates adipocytes and adipose tissue receptors, enhancing lipid metabolism and exerting a favorable influence on lipid profiles. Consequently, even with the same BF%, men may exhibit a higher metabolic risk compared to women [14]. Understanding these sex differences is crucial for gaining deeper insights into the mechanisms underlying the development of NWO and dyslipidemia and highlights the necessity of considering sex-specific characteristics in clinical interventions. Therefore, dyslipidemia management strategies should be differentiated by sex, taking into account hormonal changes, fat distribution patterns, and metabolic processes.

According to previous studies, age and sex alone can account for 49% of the variability in BF% [38]. Therefore, age and sex should be considered when assessing NWO. This study confirmed that the impact of NWO on dyslipidemia varies according to age and sex. In particular, men under the age of 65 were identified as a high-risk group requiring intensive lipid management, highlighting the need for early recognition of NWO-related health risks and the development of proactive, targeted nursing strategies. Thus, the need for early detection and management of dyslipidemia in men under 65 years with NWO is emphasized in clinical nursing practice. Personalized health counseling focusing on promoting physical activity, improving dietary habits, and supporting smoking cessation should be actively provided, alongside the development of structured lifestyle intervention programs specifically tailored for younger men. Regular lipid monitoring should also be incorporated to ensure sustained risk reduction for dyslipidemia and cardiovascular diseases associated with NWO. Moreover, in settings where younger men are concentrated, such as universities, the military, and workplaces, practical strategies to enhance accessibility to body composition assessments should be explored to support systematic health management [39].

Beyond this high-risk group, early screening of all individuals with NWO and the implementation of comprehensive nursing strategies are essential across various areas of clinical practice. Through counseling and education, awareness of NWO should be increased and motivation for self-management should be enhanced [40]. In particular, promoting behavioral changes to lower blood lipid levels is important to reduce the risk of dyslipidemia and cardiovascular complications associated with NWO. Core nursing interventions should include the encouragement of regular physical activity and dietary modifications [40,41], supported by the establishment of systematic monitoring frameworks to maximize intervention effectiveness. Furthermore, individuals should be encouraged to perform regular self-assessments and use the results to develop and refine personalized health management plans, thereby promoting sustainable health behavior changes over time.

This study sought to investigate the association between obesity type and dyslipidemia using analyses stratified by age and sex. Nevertheless, this study presents several limitations. Firstly, because this study employed a cross-sectional approach, it limited the ability to establish definitive cause-and-effect links. Secondly, in contrast to BMI, the criteria for BF% vary across studies, making direct and accurate comparisons difficult. Thirdly, by controlling for various health behaviors and other factors that may vary by age and sex, it did not examine the interaction effects among those variables, which may have limited the interpretation of their combined associations with dyslipidemia.

## CONCLUSION

This study was conducted using the 1st year (2022) 9th KNHANES data, with the aim of examining the association between obesity type and dyslipidemia in adults aged 19 and older, stratified by age and sex.

Collectively, the outcomes of this research indicate that NWO is notably connected to an elevated risk of dyslipidemia compared with NWNO, and this risk is particularly pronounced in men and under the age of 65 with NWO. This study is significant in that it conducted stratified analyses based on age and sex and identified NWO as an important high-risk group even within the normal weight range, thereby highlighting the necessity of early screening and tailored nursing interventions according to obesity type, age and sex.

Based on the above conclusions, the following recommendations are proposed. First, longitudinal studies should be performed to examine the prevalence of dyslipidemia following the implementation of obesity management programs in individuals with NWO. Second, further research should be conducted to accurately identify the key influencing factors according to sex and age and to develop standardized screening tools and management guidelines applicable to clinical nursing practice.

## Figures and Tables

**Table 1.** Differences in the Prevalence of Dyslipidemia According to Demographic, Health Behavioral, and Chronic Disease-related Characteristics (N = 3,805)

Variables	Categories	n (%)	Dyslipidemia		χ^2^	*p*
			Yes (n = 1,812)	No (n = 1,993)		
			n (%)	n (%)		
Obesity type	NWNO	1,312 (34.5)	435 (29.2)	877 (70.8)	244.25	< .001
	NWO	1,135 (29.8)	557 (46.3)	578 (53.7)		
	Obesity	1,358 (35.7)	820 (59.0)	538 (41.0)		
Age (yr)	< 65	2,733 (71.8)	1,159 (41.6)	1,574 (58.4)	88.63	< .001
	≥ 65	1,072 (28.2)	653 (61.2)	419 (38.8)		
Sex	Women	2,083 (54.7)	922 (40.3)	1,161 (59.7)	34.57	< .001
	Men	1,722 (45.3)	890 (49.8)	832 (50.2)		
Smoking	No	3,214 (84.5)	1,488 (43.7)	1,726 (56.3)	17.77	.001
	Yes	591 (15.5)	324 (52.6)	267 (47.4)		
Drinking	No	1,811 (47.6)	938 (48.0)	873 (52.0)	9.08	.012
	Yes	1,994 (52.4)	874 (43.1)	1,120 (56.9)		
Sleep duration (hr)	< 7	1,604 (42.2)	796 (47.4)	808 (52.6)	5.16	.068
	≥ 7	2,201 (57.8)	1,016 (43.7)	1,185 (56.3)		
Sitting time (hr)	< 5	424 (11.1)	192 (43.7)	232 (56.3)	0.42	.576
	≥ 5	3,381 (88.9)	1,620 (45.4)	1,761 (54.6)		
Aerobic	No	1,994 (52.4)	1,041 (49.4)	953 (50.6)	25.98	< .001
physical activity	Yes	1,811 (47.6)	771 (41.2)	1,040 (58.8)		
Subjective	Not bad	3,083 (81.0)	1,404 (43.0)	1,679 (57.0)	35.77	< .001
health status	Bad	722 (19.0)	408 (55.8)	314 (44.2)		
Hypertension	No	2,541 (66.8)	976 (37.0)	1,565 (63.0)	258.77	< .001
	Yes	1,264 (33.2)	836 (65.8)	428 (34.2)		
Diabetes	No	3,293 (86.5)	1,426 (41.2)	1,867 (58.8)	198.13	< .001
mellitus	Yes	512 (13.5)	386 (76.8)	126 (23.2)		

NWNO = Normal weight non-obesity; NWO = Normal-weight obesity.

**Table 2.** Relationship between Obesity Type and Dyslipidemia by Age and Sex

Variables	Model 1^†^ OR (95% CI)	Model 2^‡^ OR (95% CI)		Model 3^§^ OR (95% CI)	
		< 65 years	≥ 65 years	Men	Women
NWNO	1	1	1	1	1
NWO	1.66 (1.34~2.06)^**^	1.99 (1.56~2.54)^**^	.91 (0.60~1.40)	2.29 (1.58~3.33)^**^	1.39 (1.04~1.86)^*^
Obesity	2.20 (1.79~2.69)^**^	2.48 (1.97~3.11)^**^	1.00 (0.67~1.50)	2.72 (1.88~3.94)^**^	1.80 (1.39~2.32)^**^

OR = Odds ratio; CI = Confidence interval; NWNO = Normal weight non-obesity; NWO = Normal-weight obesity.

^†^None; ^‡^Controlled for sex, smoking, drinking, sleep duration, sitting time, aerobic physical activity, subjective health status, hypertension, and diabetes mellitus; ^§^Controlled for age, smoking, drinking, sleep duration, sitting time, aerobic physical activity, subjective health status, hypertension, and diabetes mellitus.

*p < .05; **p < .001.

## Data Availability

The data that support the findings of this study are available from the corresponding author upon reasonable request.
